# Advancing CRISPR-Based Solutions for COVID-19 Diagnosis and Therapeutics

**DOI:** 10.3390/cells13211794

**Published:** 2024-10-30

**Authors:** Roaa Hadi, Abhishek Poddar, Shivakumar Sonnaila, Venkata Suryanarayana Murthy Bhavaraju, Shilpi Agrawal

**Affiliations:** 1 Cell and Molecular Biology Program, Fulbright College of Arts and Sciences, University of Arkansas, Fayetteville, AR 72701, USA; roaahadi@uark.edu; 2 Department of Chemical, Biochemical, and Environmental Engineering, University of Maryland, Baltimore County, Baltimore, MD 21250, USA; 3 Department of Molecular Biology, Massachusetts General Hospital, Boston, MA 02114, USA; poddar@molbio.mgh.harvard.edu; 4 Department of Genetics, Harvard Medical School, Boston, MA 02115, USA; 5 Department of Chemistry and Biochemistry, University of Arkansas, Fayetteville, AR 72701, USA; sksonnai@uark.edu; 6 Department of Industrial Engineering, College of Engineering, University of Arkansas, Fayetteville, AR 72701, USA; murthy.1809@gmail.com; 7 Department of Biomedical Engineering, College of Engineering, University of Arkansas, Fayetteville, AR 72701, USA

**Keywords:** SARS-CoV-2, COVID-19, CRISPR/Cas, CRISPR-Dx, CRISPR-Tx, Gene targeting, detection methods, diagnostics, therapeutics

## Abstract

Since the onset of the COVID-19 pandemic, a variety of diagnostic approaches, including RT-qPCR, RAPID, and LFA, have been adopted, with RT-qPCR emerging as the gold standard. However, a significant challenge in COVID-19 diagnostics is the wide range of symptoms presented by patients, necessitating early and accurate diagnosis for effective management. Although RT-qPCR is a precise molecular technique, it is not immune to false-negative results. In contrast, CRISPR-based detection methods for SARS-CoV-2 offer several advantages: they are cost-effective, time-efficient, highly sensitive, and specific, and they do not require sophisticated instruments. These methods also show promise for scalability, enabling diagnostic tests. CRISPR technology can be customized to target any genomic region of interest, making it a versatile tool with applications beyond diagnostics, including therapeutic development. The CRISPR/Cas systems provide precise gene targeting with immense potential for creating next-generation diagnostics and therapeutics. One of the key advantages of CRISPR/Cas-based therapeutics is the ability to perform multiplexing, where different sgRNAs or crRNAs can target multiple sites within the same gene, reducing the likelihood of viral escape mutants. Among the various CRISPR systems, CRISPR/Cas13 and CARVER (Cas13-assisted restriction of viral expression and readout) are particularly promising. These systems can target a broad range of single-stranded RNA viruses, making them suitable for the diagnosis and treatment of various viral diseases, including SARS-CoV-2. However, the efficacy and safety of CRISPR-based therapeutics must be thoroughly evaluated in pre-clinical and clinical settings. While CRISPR biotechnologies have not yet been fully harnessed to control the current COVID-19 pandemic, there is an optimism that the limitations of the CRISPR/Cas system can be overcome soon. This review discusses how CRISPR-based strategies can revolutionize disease diagnosis and therapeutic development, better preparing us for future viral threats.

## 1. Introduction

**Figure 1:**
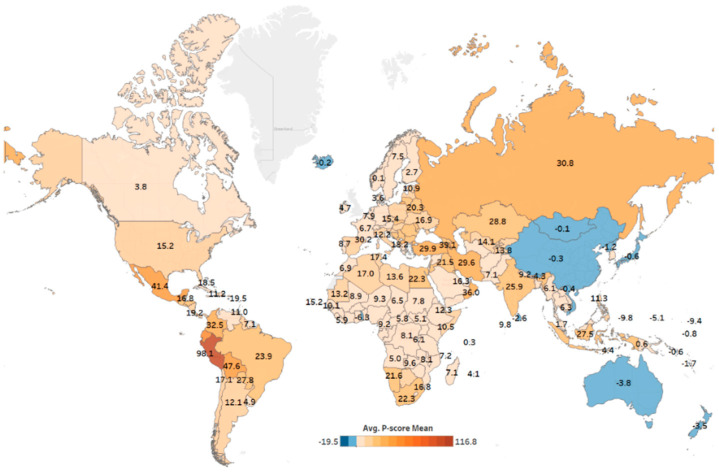
Country-wise COVID-19 death report. This figure illustrates the average P-score, representing the percentage ratio of excess mortality to expected mortality, for various countries from January 2020 to December 2021. The P-score is calculated by comparing the observed number of deaths during the pandemic to the expected number of deaths based on historical data for each country. The chart highlights differences in the impact of the pandemic across countries, accounting for both direct COVID-19-related deaths and indirect effects, such as healthcare disruptions and other pandemic-related challenges. This figure was constructed using data adopted from [17] and created using Tableau v2024.3.

The COVID-19 pandemic, caused by the SARS-CoV-2 virus, was first detected in Wuhan, China, in November 2019 [1,2,3]. The virus, known for its high infectivity and pathogenicity, rapidly spread across the globe, leading the World Health Organization (WHO) to declare it as a global pandemic [1]. This virus has since then spread globally, infecting over 775 million people and resulting in more than 7.1 million deaths as of August 2024. The pandemic’s impact was exacerbated by initial challenges in developing diagnostic tools and tracking the virus, as well as inconsistent standards for certifying COVID-19 as a cause of death, leaving global assessments of its spread incomplete. As the pandemic progressed, the SARS-CoV-2 virus mutated into several variants, including alpha, beta, gamma, delta, and omicron, further complicating efforts to control the spread [4,5,6,7]. The first significant mutation, D614G, emerged in July 2020, which was followed by alpha in the UK, beta in South Africa, gamma in Brazil, delta in the UK, India, and omicron in South Africa and Botswana (Table 1, Figure 1) [8,9].

**Table 1:** Mutations and geographical distribution of SARS-CoV-2 variants of concern. This table summarizes the notable mutations and the first detection locations of various SARS-CoV-2 variants reported as concerning.

Strains of Concern	Notable Mutations	First Detected	References
Omicron (BA.2.86)	I332V, D339H, R403K, V445H, G446S, N450D, L452W, N481K, 483del, E484K, F486P	Botswana and South Africa (Nov 2021)	[10,11,12,13,14]
Omicron (KP.3)	Q493E, F456L	Botswana and South Africa (Nov 2021)	[15]
Omicron (JN.1)	L455S	Botswana and South Africa (Nov 2021)	[16]

The first genome sequence of SARS-CoV-2 was published by the Global Initiative on Sharing Avian Influenza Data (GISAID) on 10 January 2020 [18]. The virus was originated as a previously unknown betacoronavirus with studies showing it shares 96% of its genome with a bat coronavirus [19]. On 11 February 2020, the virus was officially named SARS-CoV-2 by the International Committee on Taxonomy of Viruses, and the disease it causes was named COVID-19 by the WHO, which declared it a pandemic on 11 March 2020. SARS-CoV-2 belongs to a family of enveloped RNA viruses that infect both the upper and lower respiratory tracts [20]. Its genome contains six functional ORFs, including coding for the spike glycoprotein, envelope protein, membrane protein, nucleocapsid, and an RNA-dependent RNA polymerase [21]. The spike glycoprotein, encoded by the “*S* gene”, is crucial for the virus’s ability to infect cells [22]. This gene has been found to be phylogenetically distinct from those in other coronaviruses, although SARS-CoV-2 uses the same angiotensin-converting enzyme 2 (ACE2) receptor as the original SARS-CoV-2 virus [23]. COVID-19 symptoms range from throat pain, fever, and body aches to more severe cases involving respiratory distress, hypoxia, and death [20].

The rapid development of diagnostic tools has been critical for controlling the pandemic. Techniques range from immunological tests, like RAPID and ELISA, to molecular methods such as qPCR, which has become the gold standard for detecting SARS-CoV-2 [24]. In addition to diagnostics, effective therapeutic interventions are also essential. Current treatments focus on antiviral drugs and immune modulators with research ongoing to develop new diagnostics and therapeutics. Advanced genetic engineering techniques, such as CRISPR, could offer potential for future innovations in disease detection and treatment.

**Figure 2:**
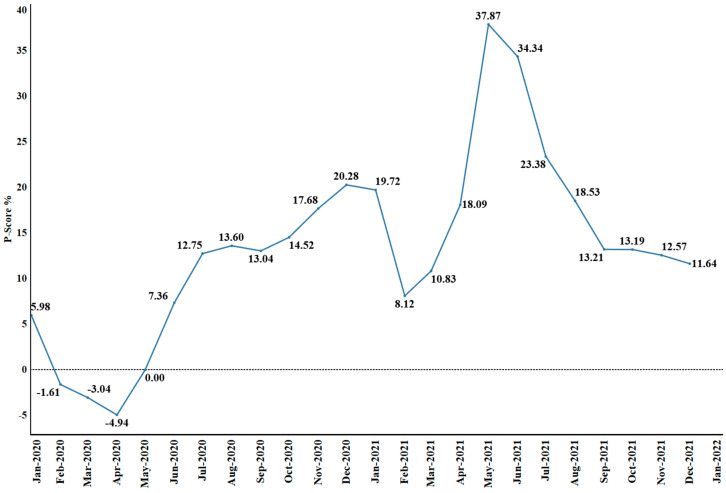
Global excess death estimates: The graph illustrates the monthly average percentage ratio of excess mortality to expected mortality, covering the period from January 2020 to December 2021. The data, sourced from Msemburi et al., 2023 reflect global trends in excess mortality during the COVID-19 pandemic [17]. This figure was created using Tableau v2024.3.

Since the first COVID-19 case, accurately assessing the virus’s impact has posed significant challenges. These challenges were further amplified when the WHO declared COVID-19 a global pandemic in March 2020. A major hurdle was the development of diagnostic tools capable of reliably detecting COVID-19. While many countries adapted existing technologies for rapid testing, others struggled with insufficient resources. Additionally, differences in how countries certified COVID-19 as the cause of death have created inconsistencies in the data, making it difficult to gauge the pandemic’s true scale. To address these issues, excess mortality (Figure 2) has emerged as a crucial metric for understanding the pandemic’s overall impact [17].

## 2. Importance of Rapid and Accurate Detection Methods

The emergence of the COVID-19 pandemic highlighted the critical need for rapid and accurate detection methods to control the spread of the virus. As SARS-CoV-2 exhibits both symptomatic and asymptomatic traits, early diagnosis is essential for implementing effective control measures and reducing the disease’s impact. Accurate detection not only aids in identifying individuals with COVID-19, including those who are asymptomatic and still contagious, but also helps monitor the virus’s spread and understand its transmission dynamics.

Diagnostic techniques have evolved to meet the demands of the pandemic, with techniques ranging from immunological tests, like RAPID and ELISA, to molecular approaches like quantitative RT-PCR (qRT-PCR), which has become the gold standard due to their high sensitivity and accuracy [25]. However, the widespread use of qPCR can be hampered by the availability of equipment and materials, leading to delays and the risk of false negatives in cases with lower viral loads [26]. As a result, the development of more accessible, time-efficient, and reliable diagnostic methods is crucial. Rapid detection methods, such as point-of-care tests and advanced molecular diagnostics, enable the timely identification of infections, which is essential for preventing further transmission and managing the pandemic effectively. Ensuring accurate and early detection remains a cornerstone of global efforts to combat COVID-19 and similar infectious diseases.

While swift detection of a virus is essential, effective therapeutic interventions are equally crucial in fighting viral diseases. Creating virus-specific treatments is a complex task due to the intricate role of host factors in the viral lifecycle, which restricts the availability of approved antiviral therapies [27]. The primary therapeutic approaches for COVID-19 focus on inhibiting the replication of SARS-CoV-2 using antiviral drugs, which are most beneficial when used early in the disease’s progression [28]. Immune modulators are also important as they help boost the immune system’s response, especially during the later stages of the infection [29]. Currently, hundreds of antiviral drugs, immune modulators, and neutralizing antibody therapies are being studied to find effective COVID-19 treatments [30]. In response to the urgent need for solutions, repurposing existing antiviral drugs has also been considered. It is equally important to explore various diagnostic and therapeutic methods to enhance our understanding and management of the disease.

In recent years, genetic engineering has made significant strides in improving disease diagnostics and treatments. CRISPR technology, which uses Cas proteins to target and break down specific genome sequences guided by RNA, has shown great potential not only as a tool for genome editing but also for developing new diagnostics, preventive measures, and treatments [31]. The precision, design versatility, and practicality of different CRISPR/Cas systems make them valuable in creating innovative approaches for diagnosing and treating viral infections, including COVID-19. Ongoing research is exploring how these technologies can transform the way we detect and treat viral diseases.

## 3. Introduction to CRISPR Technology

Clustered Regularly Interspaced Short Palindromic Repeat (CRISPR) refers to short, repeating DNA sequences found in the genomes of prokaryotes [32]. These sequences were first discovered in *E. coli* by Dr. Ishino’s group in 1987 and have since been identified in various other prokaryotes [32]. When a prokaryote is attacked by a virus, these repetitive sequences are transcribed into CRISPR RNAs (crRNAs). These crRNAs guide CRISPR-associated (Cas) proteins to target and cleave the viral RNA or DNA, effectively neutralizing the threat. This system functions as an adaptive immune response in prokaryotes [33].

**Figure 3:**
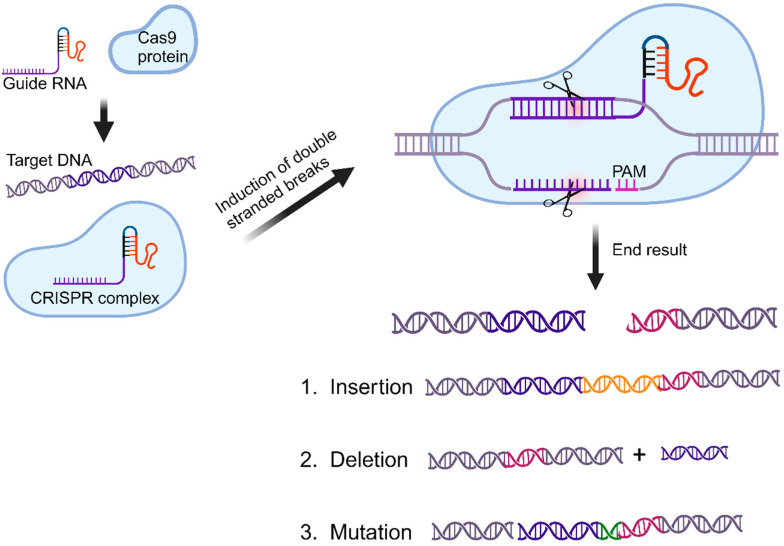
Mechanism of CRISPR/Cas9 genome editing: This process involves a gRNA that directs the Cas9 enzyme to a specific DNA sequence within the genome, where Cas9 creates a double-strand break. If a donor DNA template is supplied, homologous recombination can occur, allowing for the insertion of new genetic material at the break site. Without a donor DNA, the cell repairs the break using non-homologous end joining (NHEJ), which can introduce insertions or deletions (indels). These indels may disrupt the open reading frame of the gene, potentially leading to gene inactivation or modification. This figure was created in BioRender.

The CRISPR/Cas system is categorized into two classes, Class 1 and Class 2, with six types (I–VI) and at least 33 subtypes that continue to expand [34]. The most well-known of these is the Class 2, Type II CRISPR/Cas9 system, which has been adapted as a powerful tool for genome editing. The groundbreaking use of the CRISPR/Cas9 system as “molecular scissors” for precise genome editing led to Emmanuelle Charpentier and Jennifer A. Doudna being awarded the Nobel Prize in Chemistry in 2020 [35]. Mechanistically, CRISPR/Cas systems require two key components: a nucleic acid-binding domain that recognizes a specific DNA or RNA sequence and an effector protein (Cas) that cleaves or regulates the target nucleic acids. Since its discovery, the CRISPR/Cas system has been harnessed for a broad range of applications, including the development of advanced diagnostic and therapeutic strategies (Figure 3).

This review explores the potential of various CRISPR/Cas systems to revolutionize diagnostics and therapeutics, particularly in the context of SARS-CoV-2. CRISPR/Cas technology is already transforming diagnostic and healthcare systems. For example, the principle of “collateral cleavage activity” has been utilized in CRISPR-based diagnostics, where fluorescently labeled ssDNA/RNA reporter probes detect specific sequences [36]. Cas effectors like Cas12a and Cas13, known for their collateral activity, have become particularly popular [37]. Cas12a is effective in detecting tumor-associated viral markers such as HPV, while Cas13 excels in RNA detection for viruses like Zika and Dengue [38]. These capabilities are being leveraged to develop efficient diagnostic kits for COVID-19, as discussed in subsequent sections.

## 4. Molecular Basis of CRISPR-Based Diagnostics (CRISPR-Dx)

CRISPR-based diagnostics (CRISPR-Dx) have emerged as essential tools in molecular diagnostics, offering cost-effective and accurate detection of specific DNA or RNA sequences from infectious agents [39,40]. This technology is highly effective in the rapid detection of pathogen nucleic acids and the identification of genetic mutations, making it invaluable for diagnosing infections and detecting cancer mutations [39,41]. CRISPR-based diagnostics employs advanced signal-readout techniques to improve detection accuracy and sensitivity. The diagnostic process typically involves three main steps: target binding, signal amplification, and readout and detection.

**Figure 4:**
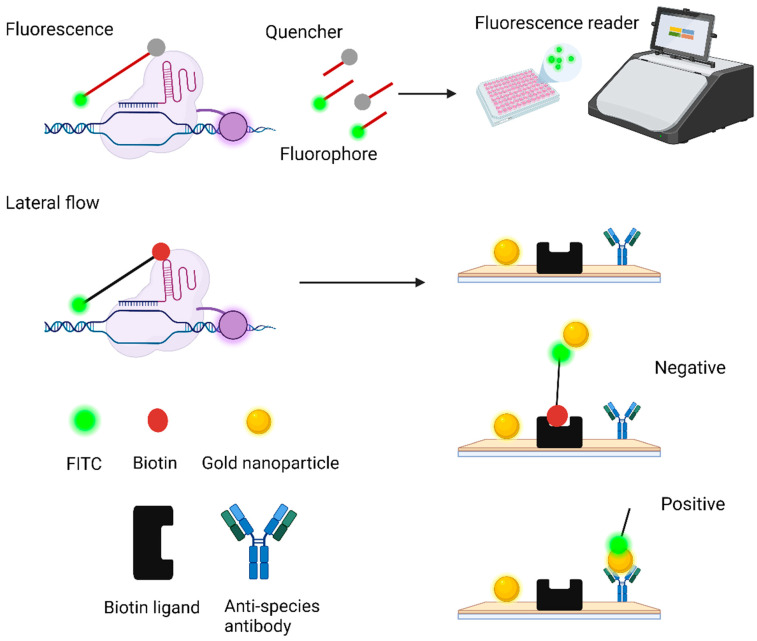
CRISPR-Cas-based detection methods using fluorescence or lateral flow. (**Top**): Fluorescence detection relies on CRISPR-Cas recognition of target DNA or RNA, which cleaves ssDNA or ssRNA linked to a fluorophore and a quencher. The released fluorophore is detected by a fluorescence reader or seen under blue light. (**Bottom**)*:* In lateral flow, CRISPR-Cas cleaves a FITC–biotin reporter. If intact, the reporter binds to a biotin ligand line on the strip, forming a single band. If cleaved, the FITC moves and binds to antibodies further down the strip, forming a second band. This figure was created in BioRender.

(a) Target Binding: The specificity of CRISPR-Dx stems from the targeted binding of Cas proteins to specific nucleic acid sequences facilitated by a guide RNA (gRNA). The gRNA is engineered to complement the target DNA or RNA sequence, directing the Cas protein to bind exclusively to the intended sequence (Figure 4). Various Cas proteins, such as Cas9 for DNA and Cas12 and Cas13 for both DNA and RNA, are used depending on the nucleic acid targeted [42,43,44]. This precise interaction between the Cas–gRNA complex and the target sequence minimizes off-target effects, enhancing the accuracy and reliability of the diagnostics. Such targeted mechanisms are crucial for effective detection and analysis in various diagnostic applications [45,46,47].
(b) Signal Amplification: Signal amplification in CRISPR-Dx is crucial for enhancing sensitivity and ensuring reliable detection. Techniques such as PCR and loop-mediated isothermal amplification (LAMP) are employed to increase the quantity of target nucleic acids, making more sequences available for detection. Additionally, certain Cas proteins like Cas12 and Cas13 exhibit collateral cleavage activity [48,49]. This activity involves cleaving adjacent reporter molecules upon binding to their specific target sequence, thus generating a detectable signal. For example, when Cas12 binds to its target DNA, it also cleaves nearby single-stranded DNA (ssDNA) reporters, producing a fluorescent signal [50]. These amplification strategies significantly enhance the sensitivity and reliability of CRISPR-Dx, making it an effective tool for various diagnostic applications [48,49,51,52,53].
(c) Readout and Detection: The final step in CRISPR-Dx involves the detection of the amplified signal with various methods tailored to specific applications. Each method offers unique advantages in terms of sensitivity, speed, and practicality, making them suitable for different diagnostic settings (Figure 4).

Fluorescent Reporters: Fluorescent reporters are widely used due to their high sensitivity and precision. When Cas proteins like Cas12 and Cas13 recognize and cleave the target sequence, they also trigger the collateral cleavage of nearby fluorescent molecules [45]. This cleavage releases a signal that can be detected using standard fluorescence equipment. This method excels in laboratory environments but can also be adapted for point-of-care settings where sensitive and rapid detection is required [45,54]. However, it may require more sophisticated equipment compared to other methods.

Lateral-Flow Assays (LFAs): LFAs are valued for their simplicity and portability. In this method, CRISPR-Cas components are integrated into a paper-based platform [53]. Upon detection of the target nucleic acid, the collateral cleavage activity leads to a visual change, such as a colored line, similar to a pregnancy test [55]. LFAs are particularly advantageous in resource-limited settings and field diagnostics, as they require minimal equipment and expertise [56]. While they provide qualitative results, they may not be as sensitive or quantitative as fluorescent reporters.

Electrochemical Sensors: Electrochemical sensors detect changes in electrical signals upon target recognition and signal amplification. These changes are typically monitored by electrodes that measure alterations in current, voltage, or impedance caused by binding events [57]. This method offers a quantitative readout and is compatible with microfluidic systems and digital interfaces, making it ideal for automated, precise, and real-time detection. Electrochemical sensors are beneficial in settings requiring high throughput and integration with digital systems, but they might be more complex to operate than LFAs [58].

A comparative overview of different methods reveals that fluorescent reporters offer the highest sensitivity, although they may require specialized equipment. Lateral flow assays (LFAs) provide a portable and cost-effective solution, making them best suited for field or resource-limited settings; however, they may lack the precision of lab-based methods. Electrochemical sensors deliver quantitative results and are compatible with automated systems, making them ideal for settings that prioritize precision and scalability [59].

## 5. Applications for Molecular Detection via CRISPR-Dx

CRISPR-Dx has demonstrated remarkable versatility and efficiency, significantly enhancing diagnostic capabilities across various fields [60]. Below are some key applications along with a description of how CRISPR is specifically used or modified for these applications:

(a) Rapid Detection of Pathogens: CRISPR-Dx platforms are highly effective in the rapid detection of viral pathogens, employing innovative technologies such as SHERLOCK (Specific High-sensitivity Enzymatic Reporter unLOCKing) and DETECTR (DNA Endonuclease Targeted CRISPR Trans Reporter) [61,62]. In these systems, CRISPR-associated proteins (like Cas13 for SHERLOCK and Cas12 for DETECTR) are designed to recognize specific nucleic acid sequences of pathogens (Table 2). SHERLOCK was developed by the Broad Institute and Feng Zhang’s team in collaboration with Sherlock Biosciences and DETECTR was developed by Mammoth Biosciences [50,63]. Upon binding to their target RNA or DNA, these Cas proteins activate a collateral cleavage activity that can be harnessed to cleave reporter molecules, leading to a detectable signal [64]. This mechanism was crucial during the COVID-19 pandemic for the rapid detection of SARS-CoV-2, providing accurate results essential for controlling the virus’s spread [65]. Additionally, CRISPR-Dx is adept at identifying bacterial and fungal pathogens, leveraging the same Cas protein mechanisms to diagnose a broad spectrum of infections and improve patient outcome [66,67,68].

(b) Identification of Genetic Mutations: CRISPR-Dx serves as a powerful tool for detecting genetic mutations, including single nucleotide polymorphisms (SNPs) and other variations linked to genetic diseases like cancer [72]. This application typically involves engineering gRNAs that are complementary to the target mutation sequences. The Cas9 protein, guided by these gRNAs, creates double-strand breaks at specific genomic locations, allowing for the detection of these mutations either by amplifying the surrounding DNA and sequencing it or by utilizing methods such as CRISPR-based amplification [72,73]. This enables early diagnosis and supports the development of personalized treatment strategies, which are particularly impactful in hereditary diseases and oncology, where early intervention can significantly alter patient outcomes [74].
(c) Environmental and Food Safety Monitoring: CRISPR-Dx is also utilized in monitoring environmental samples and food products for pathogen contamination, which is vital for public health. This involves customizing CRISPR systems to target specific sequences associated with harmful microorganisms [75]. For instance, by employing Cas proteins in a detection format similar to that used in pathogen detection, researchers can identify contamination in food products and environmental samples rapidly [76]. The specificity of CRISPR, combined with its ability to work with various sample types, enables the reliable identification of pathogens, thus helping to prevent foodborne illnesses and mitigate environmental hazards. This enhances overall safety and health standards [77,78].

## 6. Advantages over Traditional Methods

CRISPR-Dx offers several advantages over the traditional gold standard method of qRT-PCR, enhancing modern molecular detection capabilities. CRISPR-Dx is suitable for point-of-care (POC) testing for COVID-19 diagnosis due to its rapid detection capabilities, ease of use, and potential for portability. Unlike conventional methods like PCR, which require expensive thermal cyclers, CRISPR-Dx employs a straightforward molecular mechanism to detect viral RNA, generating results in as little as 30–60 min. Additionally, CRISPR-Dx can be integrated into compact, low-cost devices that do not require sophisticated equipment, making it ideal for decentralized testing in clinics, airports, and remote areas. Its high sensitivity and specificity, comparable to PCR, ensure that it can reliably identify even low viral loads, which is vital for early detection. The collateral cleavage activity of Cas enzymes amplifies the signal, enhancing sensitivity to levels that can be comparable to or even exceed those of PCR. CRISPR-Dx systems can be designed to detect multiple targets simultaneously, allowing for the detection of different SARS-CoV-2 genes or even different respiratory pathogens in a single test. Furthermore, the potential for visual readouts, such as lateral flow assays, allows CRISPR-Dx to be user-friendly and accessible in resource-limited settings. These characteristics make CRISPR-Dx a promising technology for POC testing, potentially bridging the gap between highly sensitive but lab-bound RT-PCR tests and less sensitive but rapid antigen tests.

(a) Speed: CRISPR-Dx is notably faster than traditional methods, streamlining the diagnostic process. Unlike qRT-PCR, which requires multiple steps including sample preparation, nucleic acid extraction, amplification, and analysis, CRISPR-Dx often demands less extensive sample preparation [41]. The integration of Cas proteins with gRNAs enables the direct detection of target sequences from crude samples, reducing the need for time-consuming nucleic acid extraction and purification. This approach simplifies sample preparation, making it more efficient for rapid diagnostics and point-of-care testing [79]. Moreover, many CRISPR-Dx platforms use isothermal amplification techniques like loop-mediated isothermal amplification (LAMP), which rapidly amplify nucleic acids at a constant temperature, eliminating the need for the thermal cycling required in PCR. The readout phase, using techniques such as fluorescence or lateral flow assays, typically completes within minutes, reducing the overall diagnostic time and facilitating quicker clinical and field decision making [76,80].
(b) Sensitivity and specificity: CRISPR-Dx is highly sensitive and specific, which are crucial attributes for accurate molecular detection. The collateral cleavage activity of Cas12 and Cas13 proteins amplifies the signal from low-abundance targets, enhancing sensitivity to levels often exceeding those of PCR [45]. Specificity is driven by the gRNA, which directs the Cas proteins to their target sequences with high precision. This specific targeting reduces the likelihood of false positives unlike PCR, where non-specific amplification can occur [45,81,82].
(c) Accessibility: CRISPR-Dx platforms are user-friendly and accessible, making them suitable for a broad range of users, including those in resource-limited settings. Simple protocols, pre-designed kits, and the development of portable devices such as lateral flow assays and microfluidic platforms facilitate on-site testing without specialized laboratory equipment [83]. This is invaluable for point-of-care (POC) and field testing, where rapid and accurate diagnostics are essential. Additionally, the streamlined workflow and reduced reliance on complex equipment make CRISPR-Dx more cost-effective compared to traditional PCR methods, promoting wider adoption, especially in areas with limited access to advanced diagnostic tools [84].

These benefits of CRISPR-based diagnostics, including increased speed, enhanced sensitivity and specificity, and greater simplicity and accessibility, position CRISPR-Dx as a transformative tool in molecular detection with significant potential to revolutionize medical diagnostics and public health monitoring.

## 7. Challenges and Limitations of CRISPR-Dx

CRISPR-Dx represents a transformative approach to detecting nucleic acids, offering high sensitivity, specificity, and rapid results. However, like any emerging technology, it faces several challenges and limitations:

(a) Technical Challenges: Although the CRISPR systems are highly specific, there is still a risk of off-target binding, which can lead to false-positive results. Improving the design of gRNAs and optimizing the conditions for CRISPR activity is essential to minimize these off-target effects [41,85]. While CRISPR-Dx can work with crude samples, the presence of inhibitors and complex sample matrices can affect the accuracy and efficiency of detection. Developing robust sample preparation methods that can efficiently isolate and purify nucleic acids without extensive processing is critical [41]. However, a critical limitation is the high level of technical expertise required to operate CRISPR-based diagnostics compared to other methods. CRISPR technology demands advanced technical skills, which restricts its accessibility and usability in many diagnostic and therapeutic settings. As a result, its application may be limited not only by the complexity of the technology itself but also by the need for highly trained personnel, which could hinder widespread use in clinical and field settings.
(b) Operational and Practical Challenges: Integrating CRISPR-Dx with automated high-throughput systems remains a challenge. Developing user-friendly devices that can seamlessly integrate sample processing, CRISPR-based detection, and result readout in a single platform is essential for widespread adoption [41,86]. Additionally, its real-world applicability is constrained by operational complexities. Establishing standardized protocols and guidelines for CRISPR diagnostics is necessary to ensure consistent performance across different laboratories and applications. This includes standardizing gRNA design, reaction conditions, and readout methods. Beyond the technical and operational hurdles, the potential for widespread use in real diagnostics and therapeutics is limited by regulatory, cost, and infrastructure challenges. Gaining regulatory approval requires rigorous clinical validation, which can be time consuming and complex, further delaying its entry into the market [41].

## 8. Key Targets Against COVID-19

The SARS-CoV-2 genome provides multiple targets for CRISPR-based diagnostics due to its conserved structural and functional elements. Key regions include the spike (*S*) gene, which encodes the spike protein responsible for host cell entry, and the nucleocapsid (*N*) gene, which is critical for viral RNA packaging and replication. These regions have been extensively utilized in CRISPR-Cas12 and Cas13 platforms for their high specificity and sensitivity in detecting viral RNA. Additionally, targeting host factors involved in viral replication and immune response modulation, such as ACE2 and TMPRSS2, offer complementary diagnostic avenues. The combination of viral and host targets in CRISPR diagnostics enhances the robustness and accuracy of detecting active infection, enabling rapid and scalable solutions for COVID-19 testing.

(a) Targets within the SARS-CoV-2 Genome

Spike (S) Protein: The spike protein of SARS-CoV-2 is critical for the virus’s entry into host cells as it binds to the ACE2 receptor. CRISPR-Cas13a has been effectively used to target the RNA of the spike protein, reducing viral load in infected cells [87]. This approach has been demonstrated in studies where a CRISPR-Cas13a system was designed to target specific segments of the spike protein’s RNA, showing potential as an antiviral tool against SARS-CoV-2 [87].

Nucleocapsid (N) Protein: The nucleocapsid protein is involved in packaging viral RNA and modulating host immune responses. Targeting the N protein can disrupt these processes and enhance antiviral immunity. Previous research has shown that the N protein interacts with host proteins such as G3BP2 and TRIM25, which play roles in immune response modulation [88,89]. This interaction can be a potential target for antiviral therapy, as it suppresses the type I interferon response, which is a crucial part of the host’s antiviral defense [88,89].

Nonstructural Proteins (NSPs): Nonstructural proteins such as NSP3 (papain-like protease), NSP5 (main protease), and NSP12 (RNA-dependent RNA polymerase) are essential for viral replication and transcription [90]. These proteins have been identified as potential targets for drug development, as inhibiting them can reduce viral replication and disease severity. For instance, inhibitors targeting the papain-like protease (PLpro) have shown promise in disrupting viral replication by leveraging binding cooperation to achieve potent antiviral effects [90,91].

(b) Targeting host factors

ACE2 Receptor: The ACE2 receptor is the primary entry point for SARS-CoV-2 into human cells. While not a direct target for viral RNA detection, monitoring the expression levels of ACE2 in respiratory tissues can provide indirect evidence of viral infection [92]. CRISPR-based diagnostics can be adapted to measure *ACE2* mRNA levels, contributing to a more comprehensive diagnostic approach [92,93].

TMPRSS2: TMPRSS2 is a host protease that primes the spike protein, facilitating viral entry. Like ACE2, TMPRSS2 expression levels can serve as an indirect marker of SARS-CoV-2 infection. CRISPR-based tools can be utilized to detect changes in *TMPRSS2* mRNA, offering additional layers of information to support diagnostic findings [93].

Immune Response Genes: The host immune response to SARS-CoV-2 infection involves the upregulation of various cytokines and interferons. CRISPR-Dx can target these immune response genes to identify patterns indicative of COVID-19. By measuring the gene expression of *IL-6*, *TNF-α*, and *IFN-γ*, CRISPR-Dx can provide valuable insights into the host’s response to infection, aiding in the diagnosis and potentially guiding therapeutic interventions [92,94].

## 9. CRISPR-Based Diagnostics Against COVID-19

COVID-19-based CRISPR-Dx leverages the precision of CRISPR technology to develop rapid and highly sensitive diagnostic tools for detecting SARS-CoV-2, enabling the early and accurate identification of infections even at low viral load.

**Figure 5:**
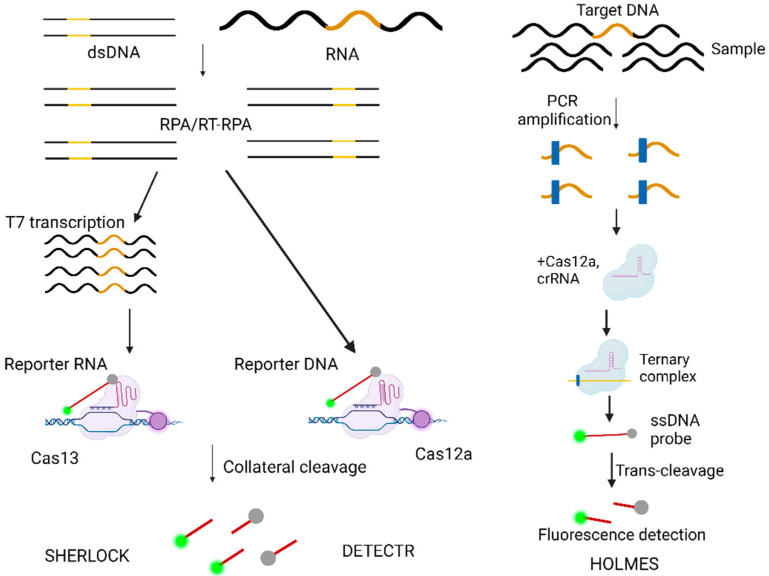
CRISPR-based diagnostic platforms for nucleic acid detection. SHERLOCK utilizes Cas13 to detect amplified target sequences, which are reverse transcribed after amplification using a T7 promoter-tagged primer. HOLMES, using Cas12, also targets amplified nucleic acids for detection. DETECTR, with Cas12, identifies amplified DNA or RNA targets through its collateral cleavage activity. All systems employ CRISPR effectors that, after target recognition, cleave corresponding reporter molecules, enabling sensitive and precise nucleic acid detection. This figure was created in BioRender.

(a) Antigen–antibody serum reactions are employed in COVID-19 diagnosis, where they detect either viral proteins (antigens) or the body’s immune response (antibodies). These tests are simple, rapid, and suitable for point-of-care use, though they tend to have lower sensitivity, particularly in the early stages of infection. On the other hand, PCR (Polymerase Chain Reaction) is the gold standard for COVID-19 detection [95], amplifying viral RNA through reverse transcription followed by DNA amplification. PCR offers excellent sensitivity and specificity, although it requires specialized equipment and trained personnel, making it less accessible in resource-limited settings.
(b) The DNA Endonuclease Targeted CRISPR Trans Reporter (DETECTR) is a diagnostic technology that uses the CRISPR-Cas12 system to detect specific DNA sequences, particularly those of pathogens such as viruses (Table 3, Figure 5). DETECTR works by combining the CRISPR-Cas12a enzyme with a guide RNA to identify and bind to a target DNA sequence [96]. Upon binding, Cas12a activates its collateral cleavage activity, cutting nearby reporter molecules and producing a detectable signal. This allows for rapid, highly sensitive, and specific detection of pathogens, making DETECTR a valuable tool for diagnosing infectious diseases like COVID-19 [97]. It is portable, cost-effective, and can be used in point-of-care settings, offering a practical solution for widespread diagnostic needs [96,97,98,99].
(c) Specific High-sensitivity Enzymatic Reporter unLOCKing (SHERLOCK) is a powerful diagnostic platform developed for the rapid, sensitive detection of nucleic acids, including viral RNA and DNA (Table 3, Figure 5) [63,82]. Leveraging CRISPR-Cas13, SHERLOCK can identify specific genetic sequences with high precision, making it ideal for detecting pathogens like SARS-CoV-2. The system uses a unique enzymatic amplification process, enhancing its sensitivity to detect even low levels of target nucleic acids [98]. SHERLOCK is designed to be portable, cost-effective, and suitable for point-of-care testing, allowing for quick and accurate diagnostics in a variety of settings, including resource-limited environments [82,96,98,99].
(d) One-Hour Low-cost Multipurpose Highly Efficient System (HOLMES) against COVID-19 is designed to quickly and affordably detect the virus (Table 3, Figure 5) [98]. It uses CRISPR-based diagnostics or LAMP technology to identify COVID-19 within one hour, ensuring high sensitivity and specificity at a low cost. Additionally, this system would be accessible in resource-limited settings, offering a scalable solution for both diagnostics and treatment. HOLMES aims to reduce transmission rates and improve patient outcomes through its rapid, multipurpose approach [98,100].
(e) The All-In-One Dual CRISPR-Cas12a (AIOD-CRISPR) is an innovative diagnostic tool designed for the rapid and highly sensitive detection of nucleic acids, such as viral RNA or DNA, including from pathogens like SARS-CoV-2 [101]. This system integrates two CRISPR-Cas12a complexes in a single reaction, enhancing the sensitivity and specificity of detection. It enables the simultaneous amplification and detection of target sequences, making it a powerful, efficient, and user-friendly tool for diagnosing infectious diseases. The AIOD-CRISPR system is also low-cost and suitable for use in point-of-care settings, offering a versatile and scalable solution for widespread diagnostic applications [101].

**Table 3:** CRISPR-Cas-based diagnostic platform.

CRISPR-Cas Type	Technique	Action Mode	Advantages	Disadvantages	Detection Method	Limit of Detection	Sensitivity
N/A	Antigen–Antibody Serum Reactions [102,103]	Detects viral antigens or host antibodies using immunoassays (e.g., ELISA) to identify the presence of COVID-19 proteins or immune response.	Rapid results (within minutes to hours) Easy to perform Suitable for point-of-care testing	False negatives/positives are more common Dependent on viral load or immune response, may miss early or asymptomatic cases	Enzyme-linked immunosorbent assay (ELISA), lateral flow assay (LFA)	Typically, nanogram to picogram levels of antigen	Lower sensitivity, especially in early stages of infection
N/A	PCR (Polymerase Chain Reaction) [102,103]	Amplifies viral RNA through reverse transcription followed by DNA amplification, enabling detection of viral genetic material.	Considered the gold standard for COVID-19 detection Detects early infection	Requires laboratory equipment and trained personnel Time-consuming (hours) Expensive Sample preparation needed	Fluorescence (via qPCR), amplification of target DNA/RNA	~1–10 copies of RNA per reaction (~10 femtomolar)	High sensitivity
Cas12a	DETECTR [62]	Utilizes CRISPR-Cas12a to bind to target DNA. Cas12a cleaves nearby reporter molecules upon activation, generating a detectable signal.	Produces results within 30 min Portable and low-cost Suitable for point-of-care	Requires nucleic acid extraction and amplification Fluorescence-based detection may need specialized equipment	Lateral flow visual readout	6.75 copies per µL of nasopharyngeal specimens	High sensitivity (100%)
Cas13a	SHERLOCK [61,63]	Uses CRISPR-Cas13 to target viral RNA. Cas13’s collateral cleavage releases a reporter signal, indicating target presence.	Portable and suitable for point-of-care Rapid detection Can detect low levels of RNA	Requires RNA extraction and amplification Needs equipment for signal detection May not be cost-effective in some settings	Fluorescence-based and lateral flow assay	42 RNA copies per reaction of nasopharyngeal swab	Highly sensitive (100%)
Cas12a	AOID-CRISPR [104]	Combines two CRISPR-Cas12a complexes in a single reaction for simultaneous amplification and detection of target DNA or RNA sequences.	Combines amplification and detection in a single reaction Low cost Minimal equipment needed for detection	Sample preparation steps still required Dual CRISPR system adds complexity Less widely validated in the field compared to SHERLOCK and DETECTR	Fluorescence-based assay	4.6 copies per µL	Ultrasensitive (100%)
Cas12a	HOLMES [105]	Uses CRISPR-Cas12 or LAMP technology for nucleic acid amplification and detection. Cas12 cleaves a reporter molecule to signal target presence within one hour.	Rapid detection within one hour Low-cost Suitable for resource-limited settings Scalable for point-of-care use	Requires amplification prior to detection Limited to specific viral RNA/DNA sequences Not as widely implemented as PCR-based methods	Fluorescence-based assay	10aM	100% sensitive

Some key advantages of CRISPR-Dx for COVID-19 therapeutics are that CRISPR-Dx can provide results within hours, enabling prompt clinical decision making. The technology offers accurate detection of the virus, reducing the risk of false positives or negatives. CRISPR-Dx could be developed for use in decentralized settings, such as clinics and homes, making testing more accessible. CRISPR-Dx can be easily adapted to detect new viral variants, ensuring its relevance in an evolving pandemic. While CRISPR-Dx has shown significant promise in pre-clinical studies, clinical trials are still ongoing to evaluate its effectiveness and safety in real-world settings. Here are some notable examples: (1) Sherlock Biosciences has developed a CRISPR-based diagnostic platform for COVID-19 detection. Their technology has demonstrated high sensitivity and specificity in pre-clinical studies and is currently being evaluated in clinical trials [63,69,70]. (2) Mammoth Biosciences utilizes CRISPR-Cas technology for diagnostics by developing a rapid COVID-19 test. Their platform is designed for point-of-care use and is currently undergoing clinical trials [50,62,71].

## 10. CRISPR-Based Therapeutics Against COVID-19

CRISPR-based therapeutics (CRISPR-Tx) explores the potential of CRISPR-based gene editing for therapeutic interventions to improve patient outcomes, such as targeting and cleaving the viral RNA genome to inhibit viral replication. CRISPR-based therapeutics (Table 4) aim to treat COVID-19 by targeting and modifying the viral genome or the host genome to prevent viral replication and disease progression [106].

(a) CRISPR-Cas13d nanotherapy is an innovative therapeutic approach designed to combat severe COVID-19 by targeting host proteases like Ctsl, which are crucial for viral entry into cells. By selectively delivering Cas13d, a smaller and more versatile CRISPR enzyme, to the lungs, this therapy specifically targets and knocks down the expression of Ctsl, hindering the virus’s ability to infect cells [107]. In animal models, lung-selective delivery of Cas13d targeting Ctsl has shown promising results in significantly reducing viral load and alleviating inflammation, making it a treatment option for severe cases of COVID-19 [106,108]. This strategy offers a targeted and precise method to mitigate the effects of the virus, reducing the severity of the disease [106,107,108].

CRISPR-Cas9, Cas12a, and Cas13a are being explored as powerful tools for antiviral therapy, particularly against COVID-19. CRISPR-Cas9 and Cas12a, which are traditionally known for their DNA-targeting capabilities, are being adapted to target the genetic material of the SARS-CoV-2 virus [107]. Although these systems primarily cleave DNA, they can be repurposed to target complementary DNA (cDNA) synthesized from viral RNA using reverse transcriptase. This allows for the precise cleavage and disruption of the viral genome, potentially halting virus replication. Additionally, CRISPR-Cas9 and Cas12a are being investigated for their ability to regulate host genes that the virus exploits for entry and replication, offering a dual approach to inhibiting viral activity. Meanwhile, CRISPR-Cas13a, which targets RNA directly, is being studied for its potential to degrade viral RNA without the need for cDNA conversion [69,107,108].

**Figure 6:**
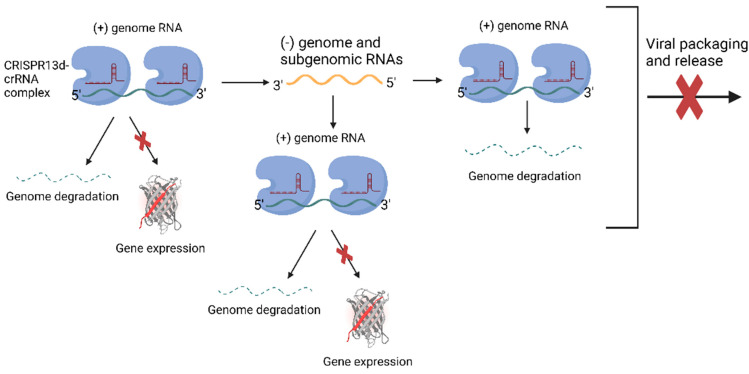
PAC-MAN is a CRISPR-Cas13d-based approach designed to counteract coronavirus in human cells. The Cas13d-crRNA complex targets and degrades the viral genome and viral mRNAs, inhibiting SARS-CoV-2 replication. This figure was created in BioRender.

(b) Prophylactic Antiviral CRISPR in huMAN cells (PAC-MAN) is a novel therapeutic approach that utilizes the CRISPR-Cas13 system to target and degrade viral RNA within human cells, providing a proactive defense against viral infections like COVID-19 (Figure 6) [109]. The PAC-MAN approach involves designing gRNAs that direct the Cas13 enzyme to cleave specific regions of the SARS-CoV-2 genome, particularly targeting conserved areas of the viral RNA to ensure effectiveness against various strains. For example, gRNAs can be designed to target the *N* gene of the coronavirus, which is crucial for viral replication and assembly. By cleaving these regions, PAC-MAN effectively prevents the virus from replicating and spreading [109]. This approach can be used as both a prophylactic (preventative) and therapeutic measure, offering broad-spectrum protection against various coronaviruses and other RNA viruses [110]. In pre-clinical models, PAC-MAN has demonstrated significant potential by effectively reducing viral loads, making it a promising tool for combating current and future viral pandemics [108].

In addition to targeting viral RNA, CRISPR technologies such as CRISPR-Cas9, Cas12a, and Cas13a are being explored as powerful antiviral therapy tools, particularly against COVID-19. CRISPR-Cas9 and Cas12a, traditionally known for their DNA-targeting capabilities, are being adapted to target the genetic material of the SARS-CoV-2 virus [107]. Although these systems primarily cleave DNA, they can be repurposed to target complementary DNA (cDNA) synthesized from viral RNA using reverse transcriptase [108]. This allows for the precise cleavage and disruption of the viral genome, potentially halting viral replication. Additionally, CRISPR-Cas9 and Cas12a can be utilized to regulate specific host genes that the virus exploits for entry and replication [69]. Notable host genes targeted by these systems include ACE2, which encodes the receptor for SARS-CoV-2, and TMPRSS2, which is a gene involved in the viral entry process. By inhibiting these host factors, CRISPR systems can provide a dual approach to suppressing viral activity [107]. Meanwhile, CRISPR-Cas13a directly targets RNA and is being studied for its ability to degrade viral RNA without the need for cDNA conversion. This specificity enhances its potential as a therapeutic agent, as it can rapidly and efficiently dismantle viral RNA, thus limiting viral replication [69].

Together, these CRISPR systems represent a versatile and promising strategy for developing antiviral therapies that could be adapted to combat not only COVID-19 but other viral pathogens.

## 11. Pre-Clinical Trials of CRISPR

Pre-clinical studies on COVID-19 necessitate the use of animal models that can be infected with SARS-CoV-2 and show a spectrum of disease severity. For this purpose, the cells in these animals must express ACE2, which is the receptor that the virus uses to enter human cells. Animals like hamsters, ferrets, African green monkeys, cynomolgus macaques [111], and rhesus macaques have ACE2 receptors that are similar to those in humans. Mice, often favored for large-scale research due to their affordability, possess an ACE2 receptor that has a much weaker affinity for the viral spike protein, resulting in only mild illness. This makes them less suitable for in-depth studies [112].

This challenge can be addressed by creating transgenic mice that carry the human *ACE2* gene. By introducing this gene into the mouse genome, these modified mice become more vulnerable to SARS-CoV-2 infection, particularly when the virus is administered intranasally. The disease progression and lung damage in these mice resemble what occurs in human COVID-19 cases [113].

Another way to produce these “humanized” mice is through CRISPR technology, which can insert the human *ACE2* gene into the mouse genome through a knock-in technique. This modification leads to ACE2 expression in various tissues, making the mice more susceptible to infection via intranasal or intragastric routes [114]. The development of these humanized mice has played a crucial role in advancing research on antiviral treatments, vaccine creation, viral transmission, disease mechanisms, and the study of severe COVID-19 symptoms.

## 12. Delivery of CRISPR/Cas Components

**Figure 7:**
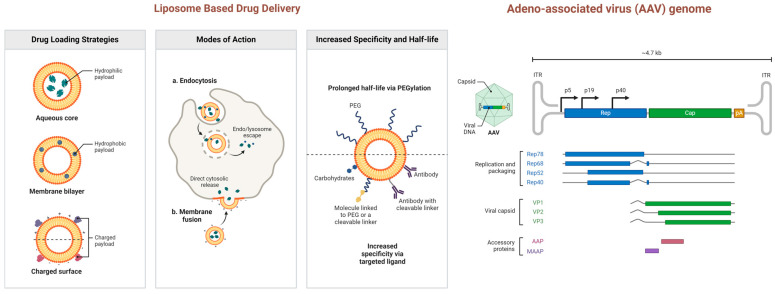
Liposome-based drug delivery and AAV genome for CRISPR/Cas component delivery. The illustration shows the use of liposomes for drug delivery, highlighting three key aspects: drug loading onto liposomes, cellular uptake mechanisms, and strategies for achieving target specificity and extended blood circulation through surface modifications. Additionally, it demonstrates the AAV genome structure, showing the key components involved in replication and packaging. It highlights the viral capsid, replication proteins, and accessory proteins necessary for AAV genome packaging and successful viral assembly. This figure was created in BioRender.

The effective delivery of CRISPR/Cas components is crucial for their therapeutic application, especially for targeting specific tissues or cells. The Cas13-crRNA system, like CRISPR/Cas9, can be delivered through various modalities, including ribonucleoproteins (RNPs), mRNAs, and plasmids. Researchers are exploring different strategies to ensure these components are delivered successfully into target cells. One promising approach is viral delivery, particularly using adeno-associated virus (AAV) as a delivery vector. The small size of the Cas13d enzyme makes it suitable for packaging into AAV, which has shown efficacy in mouse models [115]. AAV-mediated delivery is considered a viable option for in vivo applications, particularly for gene therapy (Figure 7). However, the use of AAV is not without challenges, including potential immune responses against the viral vector, which may limit its effectiveness in some cases. In addition to viral delivery, non-viral methods are also being investigated. One such method is the use of a liposome-based gene delivery platform known as High-Level Extended Duration Gene Expression System (HEDGES) [116]. This platform has been tested in immunocompetent mice and has shown promise as it does not elicit an anti-vector immune response, host toxicity, or integration of the delivered DNA into the host genome [116]. HEDGES can be used to deliver crRNAs and mRNA encoding Cas13d, offering a potentially safer alternative to viral delivery methods [36].

Another innovative approach involves the use of amphiphilic shuttle peptides, which have been shown to successfully deliver CRISPR components as RNPs to cultured human epithelial cells and mouse airway epithelia. This method, demonstrated by Krishnamurthy and colleagues, provides a non-viral option for delivering CRISPR components, potentially reducing the risk of immune reactions [117]. Synthetic carriers, such as gold nanoparticles, have also been explored for RNP-based delivery in mice models. These nanoparticles can efficiently deliver CRISPR components to target cells, offering another non-viral method that could be scaled up for clinical applications. Additionally, Guan and co-workers have demonstrated the use of poloxamine-based copolymers (peptide poloxamine nanoparticles) for delivering mRNA and plasmid DNA in vitro and in vivo, including genetic modifications in mice lungs [118,119].

Despite these advancements, the in vivo delivery of CRISPR components to specific cell types remains a significant challenge. The delivery of Cas13 components is still in its early stages of development, which is primarily because Cas13 systems are relatively new and continue to be studied and characterized. As research progresses, more effective and targeted delivery methods are likely to emerge, enhancing the therapeutic potential of CRISPR technologies.

## 13. Challenges and Limitations of the CRISPR/Cas

While the CRISPR/Cas13d system shows great promise as a tool for developing antiviral strategies against RNA viruses, including SARS-CoV-2, several limitations need to be addressed before this technology can be widely adopted in medical practice.

(a) Safe and Effective In Vivo Delivery: The most pressing challenge is the lack of a reliable and safe method for delivering CRISPR components into human respiratory tract cells. Although AAV appears to be a feasible option due to the small size of the Cas13d enzyme, the possibility of an adaptive immune response against AAV remains a concern. Exploring alternative delivery methods, such as those discussed earlier, could provide a solution for CRISPR-based antiviral delivery in humans, potentially through a nasal spray or nebulizer system.
(b) Off-Target Effects: Another significant concern is the potential for off-target effects. The specificity of crRNAs needs to be thoroughly evaluated using whole transcriptome RNA sequencing to ensure that the CRISPR system does not inadvertently target unintended sites within the genome. Reducing off-target activity is crucial for the safe and effective use of CRISPR technologies in therapeutic applications. Advanced techniques such as high-fidelity Cas9 variants (e.g., SpCas9-HF1) and gRNA design algorithms are being improved to enhance specificity and reduce these off-target impacts [120].
(c) Validation in Pre-Clinical Models: The studies conducted so far need to be validated in pre-clinical animal models, such as macaques or ferrets, to assess the specificity and efficacy of CRISPR-based antiviral strategies. Pre-clinical testing is essential to demonstrate that these technologies can work effectively and safely in living organisms before they can be considered for human use.
(d) Limitations of Current Studies: One of the most advanced studies on CRISPR-based antiviral strategies, known as PAC-MAN, was conducted on synthetic constructs of the virus rather than live SARS-CoV-2. While this study provided valuable insights, further research is needed to validate these findings using live viruses. Understanding the exact effects of the CRISPR/Cas13 system in real-world conditions is critical to its development as a therapeutic tool.
(e) Immune Response and Evasion: The Cas9 protein, derived from bacteria, can be recognized as foreign by the human immune system, potentially leading to an immune response that diminishes the therapy’s efficacy or causes adverse effects. Researchers are investigating strategies such as transient immunosuppression, the use of Cas9 proteins from less immunogenic bacterial species, or engineering humanized Cas9 variants to mitigate immune responses [64,121].
(f) Ethical and Regulatory Considerations: The potential for germline editing raises significant ethical concerns, particularly regarding the long-term effects and possible unintended consequences on future generations. Establishing comprehensive regulatory guidelines to ensure the safety, efficacy, and ethical application of CRISPR therapies is crucial. This involves rigorous clinical trials and oversight by regulatory bodies like the FDA and EMA.
(g) Viral Diversity and Mutation Rates: Viruses, particularly RNA viruses like HIV and influenza, exhibit high mutation rates, which can lead to the rapid emergence of resistant variants that evade CRISPR-mediated targeting. Designing gRNAs that target conserved regions of viral genomes can help minimize the likelihood of escape mutants [121,122].

## 14. Conclusions

Since the onset of the COVID-19 pandemic, numerous diagnostic approaches have been developed and implemented with RT-qPCR emerging as the gold standard. However, the varied presentation of COVID-19 symptoms among patients highlights the need for early and accurate diagnosis. While RT-qPCR is highly precise, it is not without limitations, including the potential for false-negative results.

In contrast, CRISPR-based detection methods for SARS-CoV-2 offer several advantages, including cost and time efficiency, high sensitivity and specificity, and the potential for point-of-care testing. CRISPR technology’s ability to target specific genomic regions within a desired genome makes it a versatile tool with applications beyond diagnostics, extending into therapeutic development. Traditional vaccines, which prime the immune system against viral proteins, may struggle to keep pace with high mutation rates in viruses like SARS-CoV-2. In contrast, antiviral therapies that target highly conserved gene sequences can potentially limit viral escape from the immune system.

The CRISPR/Cas systems provide precise gene targeting with the potential to develop next-generation diagnostics and therapeutics. The CRISPR/Cas13 system has shown promise as a highly efficient tool for targeting a broad range of single-stranded RNA viruses. The CRISPR/Cas13-based system, known as CARVER (Cas13-assisted restriction of viral expression and readout), offers the potential to be used both for the diagnosis and treatment of various viral diseases, including COVID-19. These methodologies, notably CRISPR-Dx and CRISPR-Tx, provide distinct advantages in speed, accuracy, and specificity, which are critical in managing the pandemic effectively. As these tools continue to evolve, they could substantially change the landscape of viral disease management by enhancing diagnostics precision and developing new therapeutic modalities that are adaptable to a virus’s genetic variability.

Despite these optimistic prospects, it is essential to recognize and address the inherent challenges associated with the implementation of CRISPR technologies in diagnostics and therapeutics, such as navigating the complexities of regulatory approvals as well as refining delivery mechanisms, efficacy, and safety in pre-clinical and clinical settings to maximize efficacy and minimize potential adverse reactions. While CRISPR biotechnologies may not be able to control the current COVID-19 pandemic, they hold promise for future viral threats. Overcoming the current limitations of the CRISPR/Cas system could lead to a new era in human disease diagnosis and therapeutic development, better preparing us for future pandemics and other viral challenges. 

## Figures and Tables

**Table 2:** Comparative overview of SHERLOCK and DETECTR CRISPR-based diagnostic methods.

Parameter	SHERLOCK	DETECTR
Full name	Specific High-sensitivity Enzymatic Reporter unLOCKing [63,69,70]	DNA Endonuclease Targeted CRISPR Trans Reporter [50,62,71]
Developed by	SHERLOCK Biosciences	Mammoth Biosciences
CRISPR enzyme	Cas13	Cas12
Approval	FDA EUA	FDA EUA
Detection method	Fluorescent signal via Cas13 activation	Fluorescent or colorimetric readout via Cas12
Turnaround	1 h	30 min to 1 h
Readout format	Fluorescent	Fluorescent or visual color change

**Table 4:** Comparative overview of standard COVID-19 treatments and CRISPR-based therapeutics (CRISPR-Tx).

Parameter	Standard COVID-19 Treatment	CRISPR-Tx (CRISPR-Based Therapeutics)
Mode of action	Antivirals like Remdesivir inhibit viral replication by targeting viral RNA polymerase. Monoclonal antibodies neutralize the virus by binding to its spike protein, preventing it from entering host cells. Corticosteroids reduce inflammation and immune response in severe cases.	CRISPR-based therapeutics use gene-editing tools like Cas proteins to directly target and cut viral RNA/DNA. Cas13a, for instance, can be programmed to cleave SARS-CoV-2 RNA, destroying the viral genome before replication.
Advantages	Widely available and already FDA-approved. Effective in reducing viral load and hospitalization when administered early. Broad range of options depending on disease severity (mild, moderate, severe). Readily accessible in emergency use settings. Well studied with established clinical protocols.	High specificity to the viral RNA, minimizing off-target effects. Potential for rapid adaptation to viral variants due to programmable nature. Could offer a one-time treatment that completely eradicates viral RNA. Diagnostic tool (CRISPR-Dx) has the potential for real-time, at-home detection of viral RNA.
Limitations	Effectiveness can be limited by viral mutations (e.g., antibody resistance in variants). Antiviral drugs must be administered within a specific time window to be effective. Possible side effects, including liver toxicity and immune suppression (e.g., steroids). High cost for advanced therapies like monoclonal antibodies.	Still in experimental stages with no wide-scale human trials yet for COVID-19. Delivery of CRISPR components into cells poses challenges. Long-term safety of gene-editing approaches is unknown. Regulatory hurdles and ethical concerns surrounding gene-editing technologies. Higher complexity in production and distribution.
